# All the Tau We Cannot See

**DOI:** 10.1146/annurev-med-042921-023749

**Published:** 2022-11-15

**Authors:** Bradley Hyman

**Affiliations:** Department of Neurology, Massachusetts General Hospital, Charlestown, Massachusetts, USA;

**Keywords:** Alzheimer, tau, neurofibrillary tangle, biomarker, therapeutics

## Abstract

Alzheimer’s disease (AD) was described in 1906 as a dementing disease marked by the presence of two types of fibrillar aggregates in the brain: neurofibrillary tangles and senile plaques. The process of aggregation and formation of the aggregates has been a major focus of investigation ever since the discoveries that the tau protein is the predominant protein in tangles and amyloid β is the predominant protein in plaques. The idea that smaller, oligomeric species of amyloid may also be bioactive has now been clearly established. This review examines the possibility that soluble, nonfibrillar, bioactive forms of tau—the “tau we cannot see”—comprise a dominant driver of neurodegeneration in AD.

## INTRODUCTION

The microtubule-associated protein tau is known to be involved in multiple neurodegenerative diseases including Alzheimer’s disease (AD), frontotemporal dementia, progressive supernuclear palsy, and cortical basal ganglionic degeneration. These varied neurodegenerative illnesses impact different neural circuits, and each leads to a unique (tau-containing) intracellular aggregate. Neuronal death tends to occur in the same populations of neurons where tau accumulates as fibrillar aggregates. These fibrils are perhaps most prominent in AD, where they co-occur with a second pathological lesion, β-containing amyloid plaques (Aβ plaques). The role of tau in AD is primarily discussed here, but the broader context of all the tauopathies should be kept in mind.

**Figure 1:**
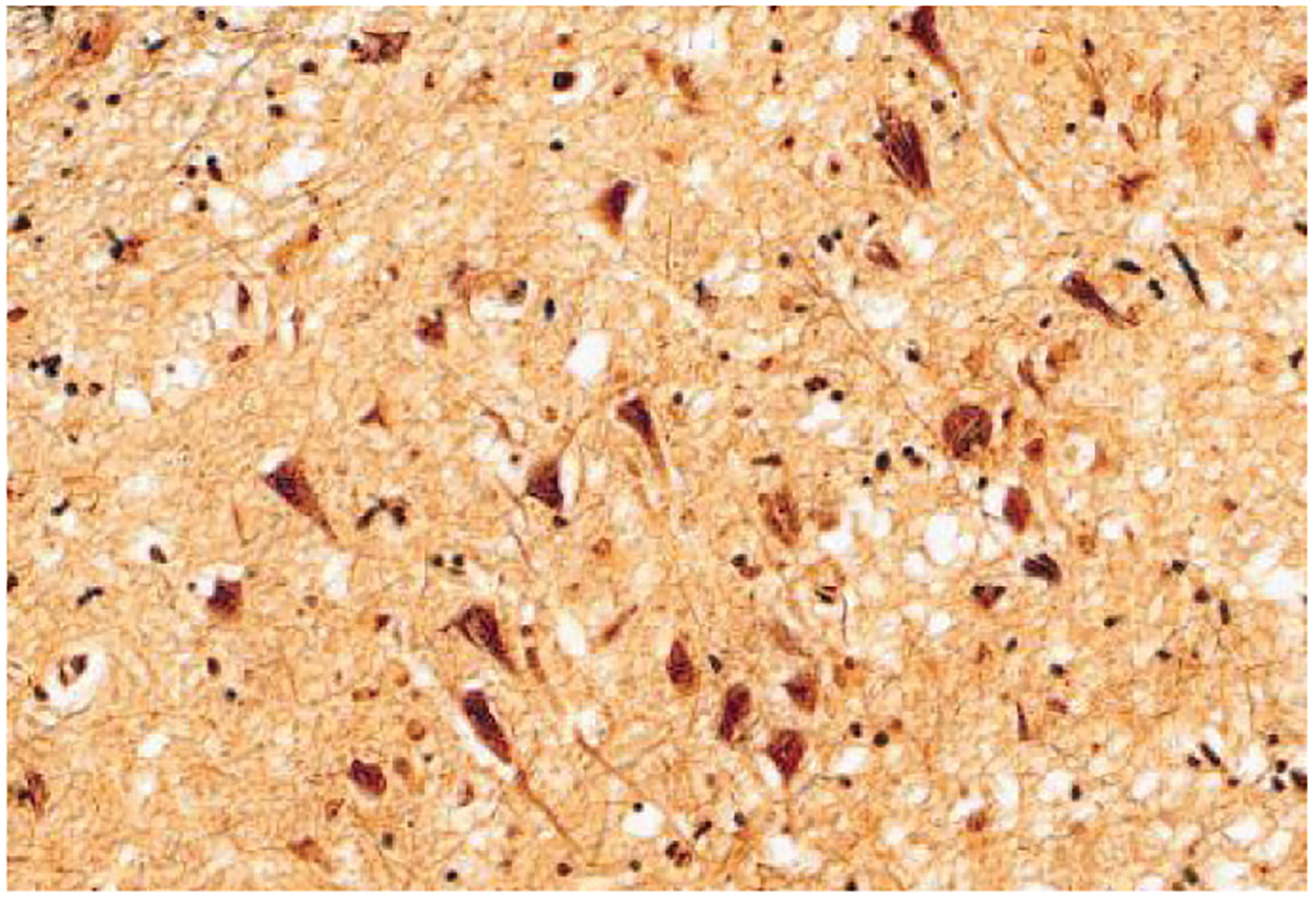
Classical Bielschowsky silver stain of neurofibrillary tangles in an Alzheimer’s disease patient.

Alzheimer himself first described these intraneuronal fibrillar lesions, which he called neurofibrillary tangles. Using a silver stain not in principle different from the stains still used in modern neuropathology laboratories (Figure 1), he observed skeins of fibrillar material that filled the cytoplasm, which represent a clear neurodegenerative phenomenon. A central dogma in AD and other neurodegenerative diseases in which tau accumulates is thus that the tau fibrils are neurotoxic. The primary diagnostic criteria for AD depend on the presence of both tau-containing neurofibrillary tangles and Aβ plaques (1, 2); the biochemical criteria similarly depend on the presence of elevated levels of phosphorylated tau (phospho-tau) (3), which is closely associated with tangles. In more recent years, tau immunostaining has supplemented silver stains to reveal an extraordinary wealth of morphologies of tau inclusions in neurons (Figure 1). Extraordinary cryo–electron microscopy studies (4–9) reveal that these aggregates have similar, but distinct, cross-β-sheet structures in different tauopathies but are consistent among patients with each unique disease. Mass spectroscopy studies show that the tau in fibrils contains dozens of abnormal post-translational modifications, dominated by a dramatic array of phosphorylation events covering over 30 sites, and also including acetylation, ubiquitination, and potentially other modifications (10, 11). The ability of classical dyes to bind to β pleated sheets ultimately led to a clinical revolution: The advent of tau PET (positron emission tomography) ligands, which permit visualization of β pleated sheets containing tau fibrils in living patients (12, 13), further cemented the clinical relevance of fibrillar tau.

By contrast with this long-held view that fibril formation is the key toxic event in AD, mounting evidence suggests that soluble, nonfibrillar tau also plays a major role both in toxicity and in propagation of tau lesions across neural systems. This tau can be detected biochemically but is not evident by classical silver stains or by electron microscopy. This review thus focuses on these other forms of tau, biochemically detectable but submicroscopic in size—i.e., the forms of tau that we cannot see (14).

## TAU FIBRILS CORRELATE WITH, BUT DO NOT NECESSARILY DIRECTLY CAUSE, NEURONAL LOSS

Tau fibrils are undoubtedly abnormal—they do not appear in normal human brain or in the brains of any other species. While phospho-tau (a hallmark of neurofibrillary tangles) can appear in some circumstances, for example in hibernating animals (15), this remains soluble and does not aggregate. Moreover, aggregates, once they are formed, appear to be long lived, perhaps lasting the lifetime of the organism. Thus, aggregated fibrils are stable, long lived, and associated with neuronal death. Why might they not be the critical toxic species?

The first concern comes from observation of neuropathological material. In Pick’s disease, where there is massive loss of cortical neurons in the frontal and temporal cortices, Pick bodies are often relatively rare—and are often in the dentate gyrus, which no doubt undergoes some neuronal loss, but not to the extent of the relatively aggregate-free cortical areas that are devastated. An analogous situation occurs in AD. While in general the pattern of anatomical locations that are affected by tangles is similar to the pattern of areas that undergo neuronal loss, there is a major discrepancy between the number of tangles and the amount of neuronal loss. Using stereological methods in patient autopsy material, Hyman and colleagues found that more than 10-fold more neurons are lost than can be accounted for by the number of tangles (16). Moreover, in rare instances of AD in which cognition is relatively preserved (resilient cases), the number of neurofibrillary tangles is the same as in typical AD patients, although the amount of neuronal and synaptic loss is minimal (17). This disconnection clearly points to the possibility that human neurons can be occupied by fibrils without neurotoxic consequence. While alternative explanations can be invoked—e.g., that some aggregates are shorter lived, lead to rapid cell death, and leave no trace after the cell is cleared—the most straightforward interpretation of these data is that tangles (and Pick bodies) correlate with neuronal loss but are not critical mediators of neuronal loss.

## TESTING THE HYPOTHESIS THAT FIBRILLAR TAU DOES NOT FULLY ACCOUNT FOR TAU TOXICITY IN ANIMAL MODELS

Overexpression of human wild-type tau leads to infrequent phospho-tau aggregates in mouse models. The most common rodent models use overexpression of a frontotemporal dementia (Pick’s disease)–associated autosomal dominant mutation in tau (of which there are dozens). These hyperaggregable, presumably misfolded species lead to aggregates in aged mice and are used as models of tau aggregation and fibril formation. The fibrils in these mice, if generated for example by a P301L mutation, are silver stain positive and Thioflavine S stain positive (indicating β pleated sheet structure), and they resemble neurofibrillary tangles morphologically and biochemically. One of the most commonly used models is the Tg4510 strain, developed by Dr. Karen Ashe, in which the animals develop progressive tau inclusions, neuronal loss, and cognitive impairments consequent to expression of the P301L tau transgene (18). They develop sarkosyl-insoluble tau—also seen in AD brain, but not in control brain or in young mice prior to morphological aggregate formation. Importantly, this mouse model was developed using a doxycycline-suppressive expression cassette, so that treatment with doxycycline suppresses expression of the transgene nearly completely. As the mice age, they develop innumerable neurofibrillary lesions, marked neuronal loss, and cognitive defects so severe that the mice perform on a memory task essentially at a chance level. Intriguingly, suppression of the transgene at that point leads to a striking change in phenotype: The tangles remain unchanged in number (for at least 6 weeks), neuronal loss essentially ceases, and the cognitive performance of the animals improves remarkably. Thus, the presence of the tau fibrils, per se, does not lead to ongoing neuronal death or cognitive deficit. Instead, suppressing the soluble forms of tau being made by the transgene seems key to improving the neuronal health and integrity of neural circuits in this model.

Analogous data come from evaluating neural system function using physiological and molecular readouts (19–22). From assessments of immediate early gene expression in the hippocampus after memory formation, it again appears that soluble tau, rather than fibrillar tau, seems to impact the responsiveness of neurons to environmental stimuli. In another example of this same principle, calcium imaging using multiphoton microscopy reveals suppressed network activity in the presence of tau overexpression (in this case either wild-type tau or P301L tau), independent of fibril formation (23, 24). Tau-induced suppression of neural networks can be relieved by turning off expression of the transgene, leading to rapid restoration of function in a time frame consistent with the half-life of soluble tau protein, not fibrillar tau. Finally, reduction of tau mRNA using zinc finger gene therapy approaches (in mice with wild-type endogenous levels of tau) leads to improvements in behavior and morphological indices of neuronal damage in the context of Aβ deposits (25), consistent with the idea that soluble rather than insoluble species lead to the neuronal phenotype.

## WHAT DOES SOLUBLE (MISFOLDED?) TAU DO THAT CAN HARM NEURONS?

**Figure 2:**
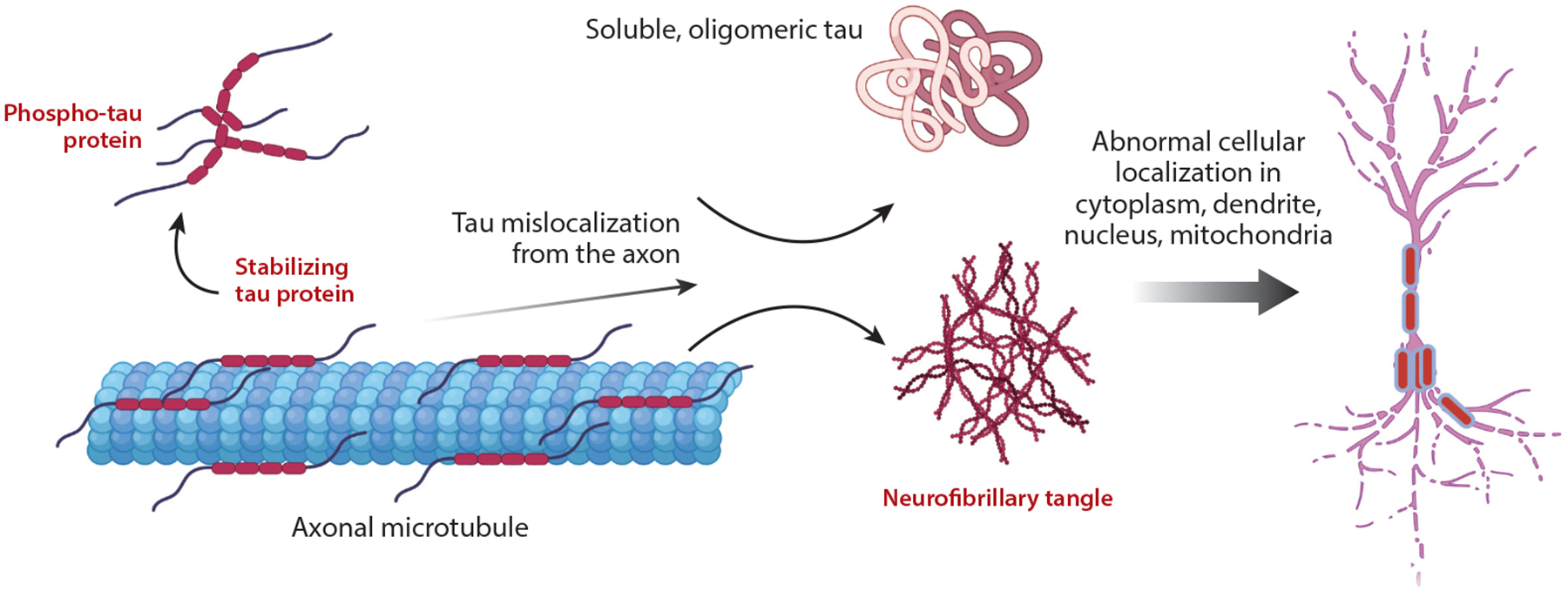
Tau is associated with microtubules. After phosphorylation and dissociation, it mislocalizes from the axon to the cell body and forms bioactive oligomers that can, as an end product, deposit as a fibril. Figure adapted from images created with BioRender.com.

Tau is a microtubule-associated protein, present predominantly in axons in mature neurons. Tau is thus assumed to stabilize microtubules, and it likely has a role in axonal transport. It is well established that tau phosphorylation, especially at specific sites that overlap with the sites that are phosphorylated in AD, diminishes the affinity of tau for microtubules (4, 26–29). For many years, a conceptual framework of the abnormality in AD could be summarized as follows: Tau becomes aberrantly phosphorylated, is less associated with microtubules, and thus can mislocalize from the axon to the cytoplasm and dendrites (Figure 2). This mislocalization can be viewed as a primary lesion, with subsequent loss of function (it no longer stabilizes axonal microtubules) and gain of function (it interacts with cytoplasmic constituents that it normally would not interact with). This simplified model underlies numerous experiments in the field and largely is supported (26, 30–42). Importantly, this model does not explicitly invoke fibril formation, which can be viewed as one more consequence of tau mislocalization to the cytoplasm.

## NUMEROUS ALTERATIONS IN NEURONAL PHYSIOLOGY HAVE BEEN ASCRIBED TO OVEREXPRESSED OR MISFOLDED TAU

Mitochondrial abnormalities, synaptic loss and dysfunction, altered axonal transport, and activation of cell death pathways (including caspase activation and inflammatory responses) have all been directly or indirectly implicated as consequences of tau mislocalization or oligomerization (20, 40, 43–52). While distinguishing cause and effect is complex, the data support the notion that fibril formation, per se, is not necessary for these effects. For example, through direct visualization of the cortex of Tg4510 P301L mice by multiphoton microscopy, Hyman and colleagues were able to determine that caspase activation, and neuronal death, appeared in many instances to precede tangle formation. Indeed, tangles forming did not lead to neuronal death over the course of the several-month imaging period (43, 51). Another example of tau-related lesions, the dystrophic neurites around plaques, also implicates the role of soluble rather than fibrillar species since the resolution of soluble forms of tau is sufficient to lead to normalization of the morphology (25, 53).

The interaction of tau with the nucleus is another example of mislocalization of the protein leading to aberrant physiology. Not only is there clear morphological evidence of nuclear alterations in AD, but also, in vitro studies using soluble phospho-tau oligomers as a perturbant find substantial alterations in nuclear–cytoplasmic transport (e.g., 31, 33, 37, 54). This is especially interesting in that other neurodegenerative diseases, including Huntington’s disease and amyotrophic lateral sclerosis (associated with mislocalization of C9orf and TDP43, respectively) have been reported to also have misfolded protein–induced alterations in nuclear cytoplasmic transport (55).

## WHICH FORM OF TAU IS IMPORTANT FOR PROPAGATION?

Misfolded, oligomeric tau is known to be secreted from neurons and taken up by recipient cells, engaging in a prion-like life cycle of inducing normal tau proteins to adopt an abnormal conformation and leading to amplification of tau across neural systems. In mice (56) and patients (57), this propagation phenomenon can take years, suggesting a complex set of mechanisms. Nonetheless, the framework of tau release, uptake, escape from degradation pathways, and induction of new oligomeric/aggregated forms is now beginning to come into focus. For example, the multifunctional receptor LRP1 appears to be a primary mechanism subverting uptake of soluble extracellular tau (58, 59), so that it can at least partly escape degradation and be released to the cytoplasm. The identification of LRP1 as a tau receptor is especially interesting because LRP1 had previously been implicated as a coreceptor for the amyloid precursor protein, and it interacts with cell surface BACE and presenilin as well as being a major apoE receptor. These roles place LRP1 at the center of multiple AD-related pathways and molecules (60–65). Moreover, LRP1 is critical for trafficking these interacting proteins to the endosomal–lysosomal compartment (58, 66), which suggests that it is potentially important for trafficking of multiple AD-related molecules either to the cytoplasm or to degradation pathways.

**Figure 3:**
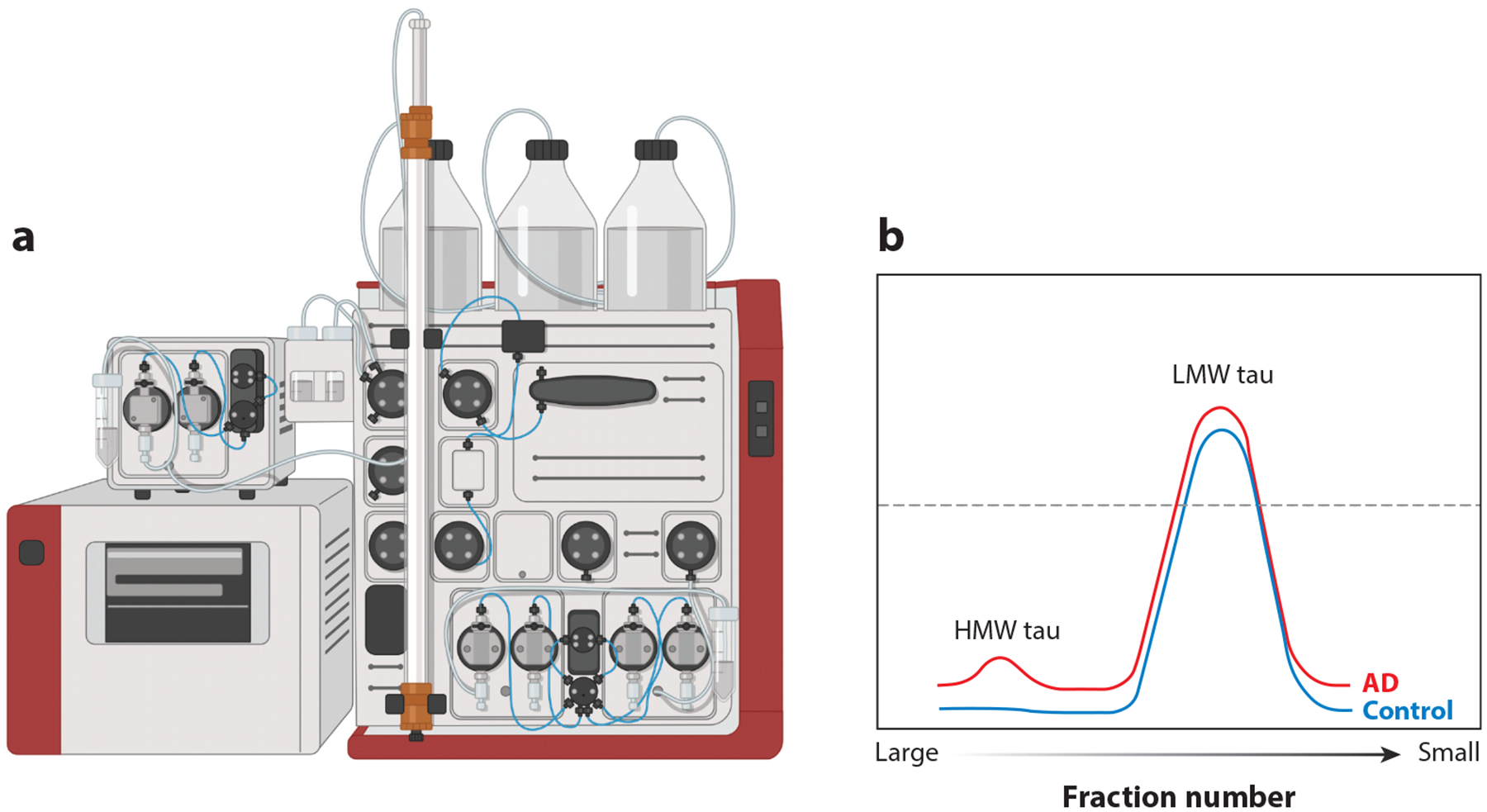
(*a*) Purification of oligomeric (high-molecular-weight, HMW) tau from human brain with Alzheimer’s disease (AD) using size exclusion chromatography. (*b*) Physiological tau runs as a single major peak at the anticipated molecular weight (low molecular weight, LMW) in both AD and control subjects; there is an additional HMW peak in the soluble fraction, which elutes in early fractions and contains all the bioactive seed-competent species. Figure adapted from images created with BioRender.com.

Soluble, oligomeric tau can also be isolated from human AD brain using simple homogenization, centrifugation, and size exclusion chromatography steps (67) (Figure 3). In carrying out these experiments, Hyman and colleagues noticed that control samples did not contain any oligomeric tau species and that the early eluting fractions contained tau that had proteopathic seed-like ability (67). Moreover, within the AD cohort, some samples seemed to contain more bioactive, propagation-prone material (per unit of tau protein) than others. In examining the clinical records, we found that those cases with the most marked propagation potential also had the fastest clinical course and had characteristic patterns of post-translational modifications (10, 11). This observation—that the characteristics of soluble tau correlate with how aggressive the clinical phenotype is—has already been replicated in two other clinical populations (68, 69). These results also reinforce the idea that soluble species of tau are important in the pathophysiology of AD.

**Figure 4:**
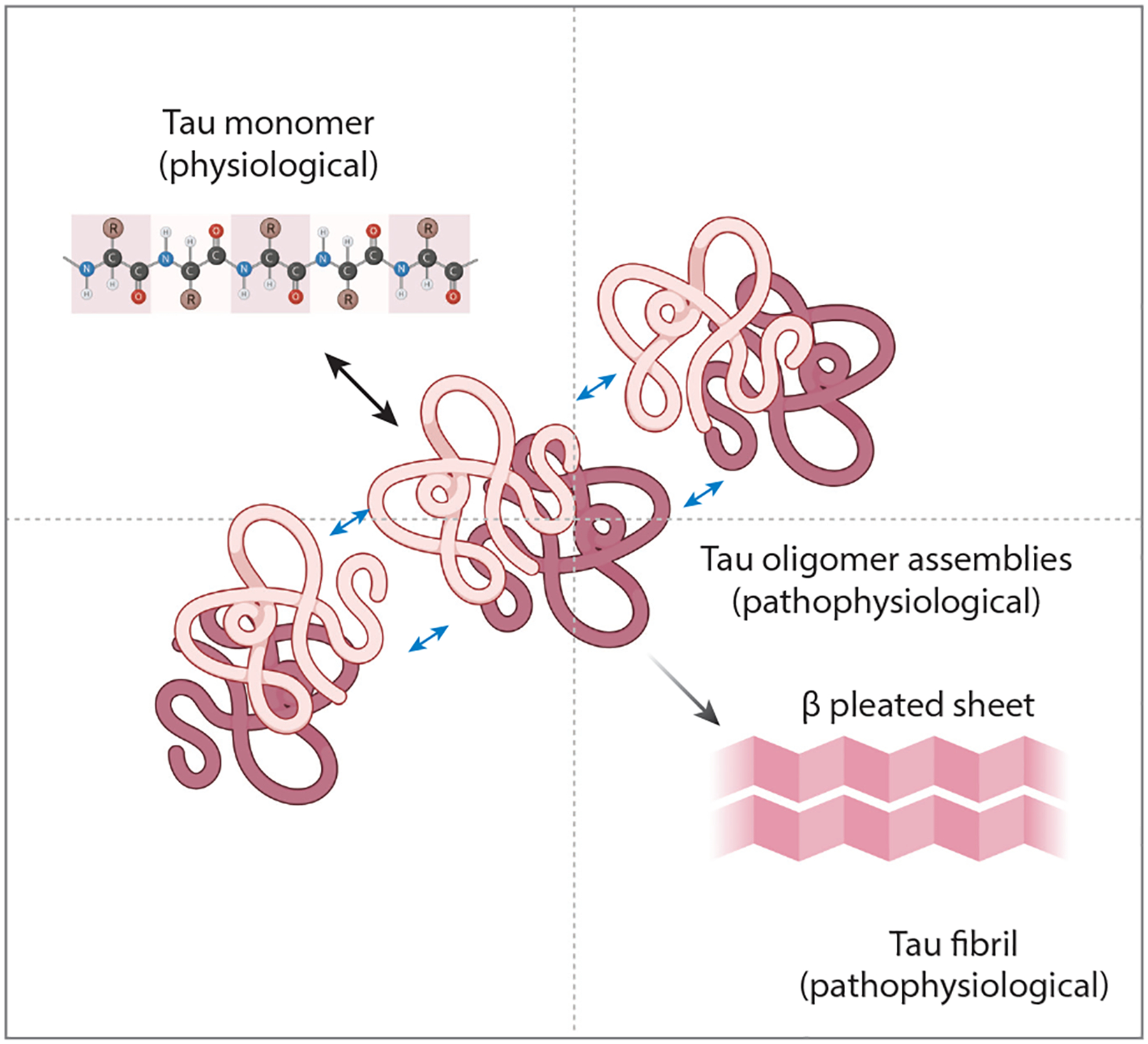
Equilibria among monomeric (physiological) tau, a population of oligomeric misfolded species, and a common fibrillar conformation. Figure adapted from images created with BioRender.com.

Hyman and colleagues suggest a model in which monomeric physiological tau is released from axonal microtubules and interacts with itself to form misfolded intermediate oligomers with multiple potential conformations [perhaps as a transient liquid–liquid phase separation phenomenon (27, 70)], which then ultimately collapse into a stable, long-lived fibril conformation (Figure 4). Our data suggest that the oligomeric forms are proteopathic seed-competent species that may well contribute to neurodegenerative phenotypes.

We speculate that the cytoplasmic misfolded tau is mislocalized—largely at the synapse, where it is in a position to be released and taken up by recipient cells in a neural system propagation model. Supporting this idea are the following observations: (*a*) synaptic preparations from amyloid precursor protein–transgenic mice contain highly phosphorylated tau species (71), oligomeric tau (72), and seed-competent species that precede the formation of frank tangles (52, 73); (*b*) in animals, tau is associated with synaptic dysfunction (24) and with a robust plasticity phenomenon, both in the context of neuritic dystrophies and as frank remodeling of degenerating neural systems (74, 75). This synapse-associated tau, which is not detected with filamentous stains or by PET ligands, is another important pool of all the tau we cannot see.

## SOLUBLE TAU AS A BIOMARKER OF NEURONAL DISTRESS IN ALZHEIMER’S DISEASE

Recent dramatic improvements in detection technology have allowed detection of tau, and even phospho-tau, in plasma from patients. Like NfL (neurofilament light chain), plasma tau has emerged as a marker of neuronal distress. It appears in several different neurodegenerative diseases, including importantly even preclinical AD patients (76–85). Interestingly, several of the diseases with high plasma tau, such as Creutzfeldt-Jakob disease, amyotrophic lateral sclerosis, and spinocerebellar ataxia, do not accumulate fibrillar tau in the central nervous system (CNS), suggesting another dissociation between the accumulation of fibrillar aggregates in the brain and a change in soluble tau biology in the CNS that is associated with neuronal distress. Caution must also be used in extrapolating impressive distinctions between AD and control subjects in studies that examined AD patients and cognitively normal controls recruited in specialty clinics to the general population. Mielke et al. recently showed that, although plasma tau 181 and 217 were elevated in AD samples, comorbidities such as chronic renal disease influenced levels as much as the presence of CNS abnormalities (86).

Although plasma tau is well established as a promising biomarker, it is not yet clear how to identify which specific tau assay from multiple competing tau platforms will turn out to be most informative, nor is it certain that the assays predict stage rather than state. Plasma tau, phosphotau 181, and phospho-tau 231 have all been measured and correlated with neurodegenerative progression in some systems, along with assays that detect an N-terminal epitope. Although it is possible that a specific epitope could be more informative than another epitope, there is no reason based on the structure of tau filaments to choose one prominent phosphoepitope over another; the relative technical qualities of the specific assays in terms of signal-to-noise ratio, stability of the epitope, and characteristics and specificity of the antibody pairs and platforms may end up mattering more than biological differences between phospho-tau 181, 231, or other species. In fact, a recent large study comparing plasma to cerebrospinal fluid tau showed that there were comparable areas under the curve (approaching 90%) for each of the phospho-tau assays measured; plasma values largely paralleled cerebrospinal fluid values (87) in distinguishing individuals with biomarker-positive AD. Another study suggested that the phospho-tau 217 assay also demonstrated favorable predictive performance in terms of replicability (88).

Plasma tau likely does give information about whether the brain contains neurofibrillary tangles, and, like early Braak stages, plasma tau elevation seems to precede overt clinical symptoms (77, 89). Plasma tau measurement could thus be used as a screening test in early-stage clinical trials to enrich trial participation with individuals more likely to have AD pathology (76, 77, 90). It appears to be more specific for AD pathology, at least compared to frontotemporal dementia and chronic traumatic encephalopathy (85, 91, 92), but it is increased in prion disease (83). Most studies do not show that the levels of plasma phospho-tau predict the rate of progression, and in a longitudinal study, plasma NfL outperformed plasma phospho-tau 181 in this regard (84). Multiple phospho-tau-based platforms are now available with additional tests in development, and there is clearly value to these tests for early detection of neurodegeneration, particularly in the context of AD. What remains to be determined is a suitable pairing between any of these tests of serum phospho-tau and a therapeutic intervention that demonstrates true activity in cognitive preservation when applied at an early stage of AD. Early trials are evolving along these lines, however, and there is hope that these early-detection tools will allow enrichment of trials with early AD patients with the potential to benefit from anti-tau antibodies or other novel therapies prior to the onset of irreversible cognitive decline.

The relevance of tau biomarkers for amyloid and other AD-directed therapeutics is intriguing. The best published data are from the Aduhelm trial (93), where a small subset of individuals was analyzed and their plasma tau levels moved “towards normal.” The clinical significance of this outcome is uncertain, since a link to clinical symptoms is not yet well enough established to justify plasma tau as a primary endpoint for a trial.

## SUMMARY

In AD, tau protein accumulates in multiple forms. The classical fibrils that were initially identified and named neurofibrillary tangles by Alzheimer over a century ago have recently been further characterized on a structural level by elegant cryo–electron microscopy work. These fibrils appear to be essentially identical from patient to patient. More recently, some work has also focused on soluble, oligomeric species that occur in the cytoplasm, can be released from neurons and taken up by recipient cells, and can induce a wide variety of potentially toxic phenotypes. In contrast to the fibrils, these oligomers (and their bioactivity as measured by propagation) vary to some extent among individual patients, and this heterogeneity correlates with rate of progression of the disease process across neural systems. These emerging data suggest that in addition to fibrillar lesions, the misfolded soluble oligomeric species—which are undetectable by classical stains that detect fibrils—may be bioactive and contribute to neurodegeneration.
